# Bee Vectoring: Development of the Japanese Orchard Bee as a Targeted Delivery System of Biological Control Agents for Fire Blight Management

**DOI:** 10.3390/pathogens9010041

**Published:** 2020-01-04

**Authors:** Neelendra K. Joshi, Henry K. Ngugi, David J. Biddinger

**Affiliations:** 1 Fruit Research & Extension Center, Entomology, Pennsylvania State University, Biglerville, PA 17307, USA; 2 Department of Entomology, 501 ASI Building, Pennsylvania State University, University Park, PA 16802, USA; 3 Department of Entomology and Plant Pathology, 217 Plant Sciences Building, University of Arkansas, Fayetteville, AR 72701, USA; 4 Fruit Research & Extension Center, Plant Pathology, Pennsylvania State University, Biglerville, PA 17307, USA; henry.ngugi@fmc.com

**Keywords:** fire blight, pollinator, bees, *Bacillus subtilis*, bee vectoring technology, biological control, apple, *Osmia cornifrons*, orchard, bacterial disease

## Abstract

Fire blight, which is caused by the bacteria *Erwinia amylovora*, remains one of the most important diseases limiting the productivity of apple and pear orchards in the United States. In commercial orchards, in-season fire blight management relies exclusively on the use of antibiotic treatments (such as streptomycin and oxytetracycline) and on bacterial biocontrol agents whose efficacy is limited. We hypothesize that the efficacy of the biocontrol agents can be greatly enhanced through targeted delivery to flowers, which serve as initial infection courts, using the Japanese orchard bee, *Osmia cornifrons*. Many factors, such as the synchrony of life cycle with plant phenology and specificity to pomaceous plants, suggest that *O. cornifrons* could be an excellent vector of the biocontrol products during bloom in pome tree fruits. However, deployment of this pollinator species to deliver biocontrol agents for fire blight control has not been attempted previously due to the lack of an efficient system to pack the bodies of the bees exiting nesting tubes with the biocontrol products. In this study, we design and test a dispenser system to facilitate the use of *O. conifrons* as a vector for commercially available biocontrol products for fire blight control. The effectiveness of *O. conifrons* to deliver biocontrol agents to flowers, and to effect secondary dissemination from treated to untreated flowers is also evaluated in greenhouse experiments. We found that the *O. conifrons* bees were able to use the nest dispenser designed for the delivery of biological control products, and are effective in vectoring and delivering the *Bacillus subtilis*-based biological control product (Serenade^®^) to apple blossoms. We also found that the *O. cornifrons* were effective in secondary inoculation of this biological control product to newly-opened flowers. These findings suggest the potential use of commercially available *O. conifrons* and other orchard bees in targeted delivery of biological control products for fire blight, and possibly other diseases, in different fruit crops.

## 1. Introduction

Fire blight disease, caused by the plant pathogenic bacteria *Erwinia amylovora*, is one of the most economically important diseases in pome fruits [1,2], limiting the production of apples in fruit growing regions of the eastern United States and many other apple and pear growing regions of the world [3,4,5,6]. It is also a severe problem in pear orchards in the eastern United States, and because of that, the production acreage in this region is kept quite small—only 3200 acres worth $10 million compared to 61,200 acres worth $291 million along the West Coast [7]. If not controlled in orchards, *E. amylovora* infects blossoms, fruits, as well as vegetative parts such as shoots and wood in host trees [8,9]. In severe infections, it can also infect rootstock crowns of fruit trees, eventually causing death [4,8]. Major changes in the apple production industry in the last 20 years have resulted from the widespread adoption of new cultivars and high-density tree plantings in commercial orchards to reduce labor costs. Recent plantings of different susceptible apple cultivars and certain dwarf rootstocks have greatly increased the risk of fire blight disease in orchards [2]. An incidence of only 10% fire blight disease in new high-density orchards with 4-year old trees is estimated to cause losses of up to USD 8400/hectare [10]. In the recent past, just a single incidence of fire blight epidemic in apple orchards in Michigan resulted in the loss of 220,000 trees with an estimated economic loss of about USD 42 million [5]. Fire blight causes heavy losses in other geographical regions as well. For example, the eradication of fire blight-infected dwarf pome orchards in Switzerland cost approximately CHF 50 million [6]. In addition, fire blight incidences may cause quarantine issues resulting in export denial in some countries.

Effective management of the fire blight disease in commercial apple orchards requires integrated approaches. Most fire blight management strategies have focused on the reduction of inoculum in the orchard (e.g., early season sprays of copper bactericides or the pruning of cankers and infected tissues), and the use of antibiotic treatments to prevent infection during the blossom blight phase [2,11,12]. Control of the blossom blight phase of the disease remains the critical component in fire blight management, and approximately 10,000 lb (~4,536 kg) of the active ingredient (a.i.) of streptomycin are applied prophylactically each year to apples and 7000 lb (~3175 kg) a.i. to pears in the US [13]. Properly timed applications of this antibiotic during the bloom period in orchards can provide over 80% control of sensitive strains of the pathogen, but resistant strains have developed in most areas of western United States and Michigan that greatly reduce its effectiveness [14,15]. Although another antibiotic, oxytetracycline, can be used in orchards for these pathogen strains [16,17], it is partially effective against these strains and there are no other registered alternatives that provide a high level of control. If fruit growers fail to control blossom blight during the spring, no control strategies are available to control shoot blight and rootstock blight during the summer months. Use of streptomycin in organic apple production, while allowed, is strictly regulated. The continued use of large amounts of antibiotics for agricultural production has raised serious concerns about developing cross-resistance in human pathogenic bacteria [18]. The need for different management tactics and alternative tools (such as the use of biological control methods or development of resistant varieties) for the management of fire blight, therefore, cannot be overstated.

Biological control of *E. amylovora* is an important alternative to conventional antibiotics in managing fire blight disease [19]. Several biocontrol products for fire blight control have been registered or are at an advanced stage of registration. These include different products based on *Bacillus subtilis, Pseudomonas fluorescens* [20,21], as well as several strains of *Pantoea agglomerans* that are at advanced stages of testing [22]. Epiphytic bacterial biocontrol agents have not consistently provided high levels of fire blight control [23,24]. In biological control, inhibition of *E. amylovora* results from the pre-emptive and competitive colonization of the near-sterile environments of the nutrient-rich flower stigma of newly opened flowers. If environmental conditions are highly favorable for multiplication or if the initial inoculum is very high, pre-emptive colonization becomes the major factor responsible for disease control [25]. Current applications of biological control-based products with airblast sprayers are unable to target the material applied directly onto the stigmatic surfaces and floral nectarines where initial pathogen build-up and subsequent host colonization begins [26]. Of the biocontrol bacteria that do reach the flowers, most never reach the stigma because of flower location/orientation or because they were not open (i.e., flowering) at the time of product application. Targeted delivery directly to the stigma and nectaries would greatly reduce both the rates and costs of bacterial biological control agents as well as improve their efficacy.

Apple growers heavily rely on bees and other flower-visiting arthropods for pollination [27,28]. The characteristics that make bees excellent pollinators also make them appropriate carriers of organically approved disease biocontrol formulations, which they can deposit directly on the stigma while foraging on open flowers. This strategy has met some success using honey bees (*Apis mellifera*) in various crops [25,29]. Many factors such as synchrony of life cycle with pome fruit phenology, crop specificity, and greater efficacy in pollinating flowers make solitary bees (e.g., the Japanese orchard bee, *Osmia cornifrons*) more efficient vectors of biocontrol products than honey bees [30,31]. *Osmia cornifrons* is commercially available as a tree fruit pollinator, and fruit growers deploy them for pollination services in their orchards. Due to parasitic mites and several other stressors, the population of honey bees has been declining in different regions [32,33,34,35,36,37,38,39]. In the recent past, such declines have greatly increased the cost of honey bee rentals to fruit growers. Alternative pollinators such as *O. cornifrons* and the native Blue Orchard bee, *O. lignaria*, are increasingly being used as replacements or supplements to honey bees in many crops [27].

The main challenge while using these solitary bees as vectors of biocontrol agents has been to devise a strategy to pack the body of the individual bees with sufficient amounts of the biocontrol agent before they exit their nesting structures. In the case of colony bees such as honey bees, this difficulty was overcome by placing the biocontrol agent in a dispenser box attached to the standard bee hive [29]. However, a different strategy is required for the solitary bees, which, by definition, do not live in large colonies like honey bees or bumble bees. Until recently, such a strategy had not been available, but research from Italy on the European Orchard bee, *O. cornuta*, suggests that it is possible to devise a dispenser that would facilitate the delivery of biocontrol agents using other solitary bees such as the Japanese orchard bees [40,41]. In this context, the objectives of the current study are to (1) design a vector dispenser suitable for the Japanese orchard bee (*O. cornifrons*), (2) determine if the *O. cornifrons* bees using the dispenser can vector the biocontrol product, and (3) determine the ability of *O. cornifrons* bees to secondarily transmit biocontrol bacteria between flowers.

## 2. Materials and Methods

This study consisted of three experiments on bee vectoring of a biological control agent for fire blight management. Experimental procedures for each of these studies are summarized below.

### 2.1. Designing and Optimizing Nest Dispenser System for Osmia cornifrons

**Figure 1:**
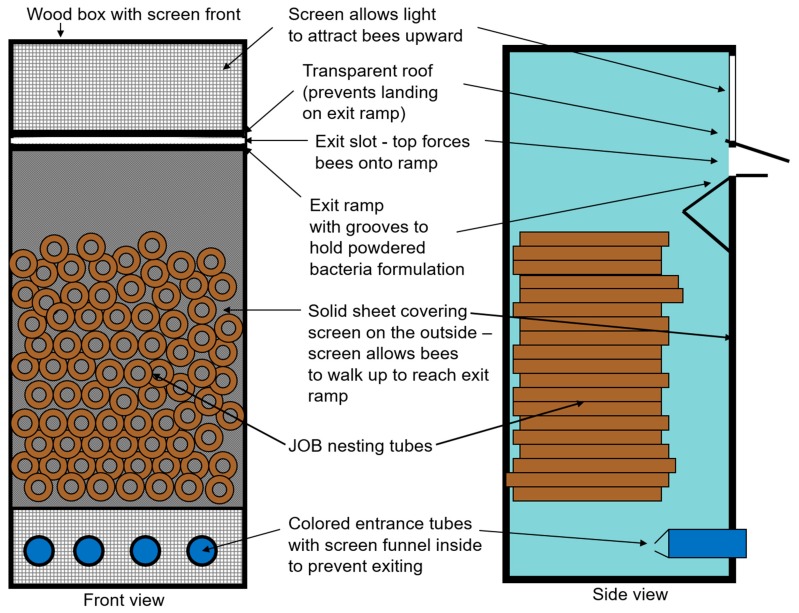
Front and side views of the dispenser nest developed for deploying the Japanese orchard bees (JOB) for vectoring biological control products.

We designed and tested a nest dispenser suitable for the *O. cornifrons* bees following a model described by Maccagnani et al. [41] for the European orchard bee, *O. cornuta*. The design is of a simple wooden structure (H,D,W: 26, 20.5, 17.5 cm) consisting of an exit ramp (made of transparent plastic) with a shallow station at the base of the ramp designed to hold the biocontrol product in a fine powder or granular form (Figure 1). We scaled up this dispenser to accommodate at least 250 female *O. cornifrons*, which are considered adequate for pollination of one acre of apples. In the dispenser system, we modified the upper exit ramp to allow for quick loading of the biocontrol product into the grooves with a small spoon. A previous similar design for a mason bee species allowed 20% of the bees to exit via the entrance holes [41], thus reducing their effectiveness as active carriers of the antagonistic bacteria. The new dispenser design was modified to increase the efficiency of using the exit slot, by covering the screened front to allow light to enter only from above, attracting the bees upward to the exit ramp. We also separated the exit slot from the entrance tubes by fitting the entrance tubes with a screen flap directing the bees upward but restricting their movement downward, all of which greatly reduced the use of the entrance tubes by exiting bees.

Based on our experience from different trails with the *O. cornifrons* bees, we color-coded the entrance holes. Bees that have been shown the color-coded entrance tubes “remember” the entrance holes of their nest at the time of their initial orientation flight, so that upon returning, they fly directly to a specific entrance hole. After constructing the dispenser system, we tested the proper use of entrances and exits in the nesting dispenser by observing all bees entering and exiting the dispenser over a 15-min period and repeated the observations three times. The observations were made with the Serenade^®^ MAX (*B. subtilis* strain QST 713) formulation (Bayer CropScience, St. Louis, MO) containing a minimum of 7.3 x 10^9^ cfu/g placed in the exit grooves of the nest dispenser. The percentage of bees utilizing the exits and entrances correctly of the dispenser nest was calculated for each trial.

### 2.2. Osmia cornifrons Vectoring of Biological Control Product Using the Dispenser

In this experiment, the amount of the biological control product collected by *O. cornifrons* bees exiting the dispenser and the proportion of the product that was deposited on individual flowers following legitimate visits was documented at the deployment rate of 250 bees/acre (25+ nest tubes) recommended for pollination. The same biological control product formulation used in Experiment 1 was used for this experiment. To determine the amount of the biocontrol product collected by an individual insect, bees exiting the dispensers were captured and placed in glass vials containing 2 mL of sterile 0.1 M potassium phosphate buffer (KPB) amended with 0.1 mL of Tween-20 surfactant per liter. The vials were shaken vigorously for 30 s, followed by incubation in a sonicating bath for another 30 s to dislodge the bacteria adhered to the body of the vectors. The wash buffer was subjected to serial dilution and plated either on nutrient yeast extract dextrose agar (NYDA) medium for *B. subtilis* [42]. All the plates were then incubated at 28 ˚C and the total number of colony-forming units (CFUs) were determined within three days of plating. Bees exiting dispensers in which no biocontrol product was placed were used as controls. Eight bees were assayed for the Serenade treatment in each of the three experimental runs. We also collected data on primary transmission. For this purpose, flowers (n = 8, from each experimental trial) were collected from potted crabapple trees (n = 6) immediately after being visited by the *O. cornifrons* bees carrying *B. subtilis* based biological control product and placed singly into vials (replicates) for assaying the quantity of *B. subtilis* by dilution plating as described above. The amount of *Bacillus subtilis* (in terms of CFUs) carried by the *O. cornifrons* bees exiting the dispenser nest was quantified in each trial.

### 2.3. Determining the Ability of O. cornifrons to Secondarily Transmit Biocontrol Bacteria between Flowers.

This experiment was conducted in two parts to examine the secondary transmission of *B. subtilis* based biological control product (Serenade^®^) by *O. cornifrons* bees. In order to isolate both the potted crabapple trees from wild bee visitation and to concentrate the test *O. cornifrons* from the nest dispensers on them, the experiment was conducted very early in the season (~3 weeks prior to normal bloom period). Early bloom development in potted crabapple trees and early emergence of overwintering *O. cornifrons* bees were facilitated by warming in the greenhouse and laboratory, respectively.

In the first part of the experiment, we measured primary transmission using potted crabapple trees and treated *O. cornifrons* nest dispensers. Crabapple trees were used rather than apples for use in the greenhouse because of the much heavier flower production on small potted trees. The potted crabapples trees (at 25–50% bloom, trial 1: n = 10, trial 2: n = 4) and the treated *O. cornifrons* nest dispensers (n = 2) were placed in an isolated grass area where there were no crops in bloom at the time. Consequently, we were able to isolate *O. cornifrons* bees on the only food source (i.e., crabapple flowers) around and observed no other bee species foraging on the crabapple trees at that time. Flowers in potted trees were closely observed and were harvested and placed in vials containing 5 mL of KPB solution immediately after *O. cornifrons* visitation. The experiment was repeated twice, and the flowers (n = 120) visited by treated *O. cornifrons* bees were collected and processed for counting CFUs of *B. subtilis* as per the protocol described in Experiment 2.

In the second part of this experiment, we measured the secondary transmission, and potted crabapple trees (i.e., primary exposure trees) that had been directly exposed to *O. cornifrons* bees carrying Serenade were immediately moved about 5 miles (~8 km) to an isolated location in a wooded area. All unopened blossoms on these trees were removed prior to transport so that only flowers that had been exposed to *O. cornifrons* with the Serenade product were left for the secondary transmission trial. Another set of potted crabapple trees at 25–50% bloom, which had never been exposed to bees or Serenade, were moved from the greenhouse and placed within 2 m of the primary exposure trees. The experiment was repeated twice (Trial 1 and 2) with a total of 10 potted trees of each treatment in the first replicate and 4 trees in the second. After 24 h of exposure to the new (untreated) *O. cornifrons* nests, flowers were collected at random from the previously unexposed trees and processed following the dilution and agar plating protocol of Experiment 2. A total of 120 flowers were collected, serially diluted 4 times and 480 plates were incubated for counting CFUs. Standard errors and means of each trial were calculated. Flowers (n = 5) collected from unexposed trees (n = 4) from the greenhouse prior to moving them to the grower field site were used as controls to make sure there was no prior contamination of the trees with the biological control product used in this experiment.

## 3. Results and Discussion

### 3.1. Nest Dispenser System for Osmia cornifrons

**Figure 2:**
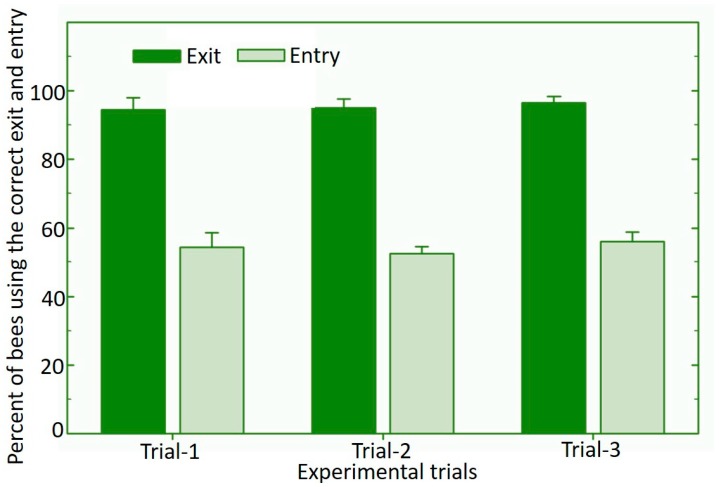
Utilization of the exit and entrance of the dispenser nest by the Japanese orchard bees. Error bars represent standard error.

The newly developed vector dispenser nest (Figure 1) was found suitable for dispensing *O. cornifrons* bees. On average, the bees utilized the right exit 95% of the time, and were exposed to the biological control product (*B. subtilis*, Serenade) while exiting from the dispenser nest. However, only about 50% of bees under observation returned via the lower entry tubes of the dispenser nest (Figure 2). The remaining bees in this observation re-entered via the exit grooves of the dispenser nest. Properly exiting the dispenser nest by *O. cornifrons* bees and their contact with *B. subtilis*-based product (while exiting) was the most important feature of the newly developed dispenser nest-box. Though almost 50% bees were entering the nest dispenser from the exit grooves, this was not a problem because the exit grooves contained the biological control product and bees picked up more of the product on their body hairs. However, the additional biocontrol product picked up by bees on the way out would depend on other factors, such as the speed of bees while exiting from exit holes of the dispenser nest. Depending on the number of bees in the nests, the dispenser system would require re-application of product into the dispensing grooves at least once a day.

### 3.2. Vectoring of Biological Control Product Using the Osmia cornifrons Dispenser

**Figure 3:**
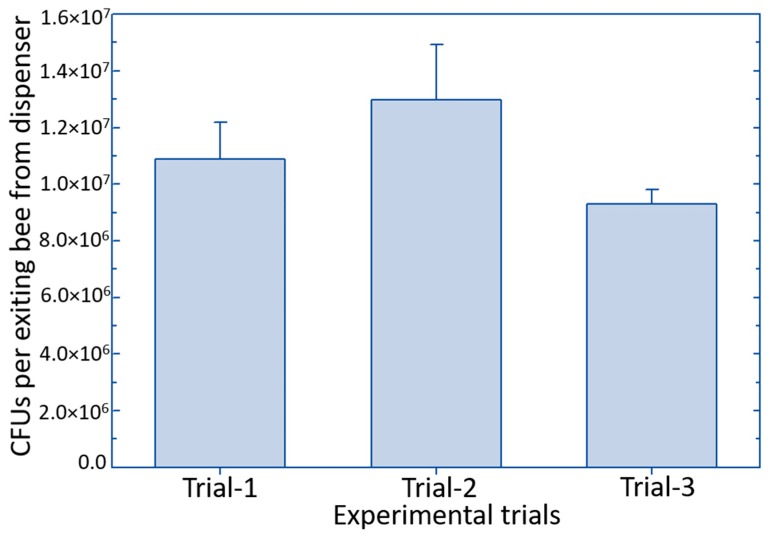
Amount of *Bacillus subtilis* (in terms of colony-forming units) carried by the Japanese orchard bees exiting the dispenser nest. Error bars represent standard error.

**Figure 4:**
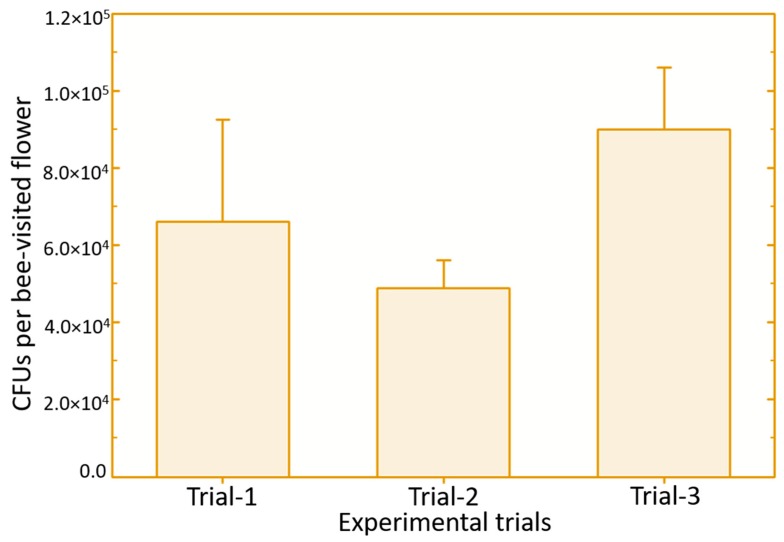
Amount of *Bacillus subtilis* (in terms of colony-forming units) deposited by the Japanese orchard bees on crabapple flowers. Error bars represent standard error.

In this study, the amount of biological control product (estimated as *B. subtilis* colony-forming units) carried by the *O. cornifrons* bees while exiting the dispenser could be considered optimal for the disease control (Figure 3). Compared to the amount of a similar biocontrol product (Serenade) carried by honey bees for the protection of blueberries from mummy berry disease [42], *O. cornifrons* bees carried approximately 20 times more product upon leaving the newly designed dispenser nest. The amount of *B. subtilis* that *O. cornifrons* bees actually delivered to the apple flowers was also high (Figure 4)—about 18 times higher than was found on blueberry flowers after delivery by honey bees in a previous study [42]. These differences could be due to many factors such as dispenser design, insect hair and exoskeleton characteristics that are difficult to identify because our study was not designed to compare the two insect vectors. Nevertheless, these data suggest that for purposes of delivering biological control products, *O. cornifrons* may be a better vector than honey bees; however, this observation requires further investigation.

The data for primary and secondary transmission of the biological control product in this study using *O. cornifrons* is presented in Table 1. Compared to another similar bee species (*O. cornuta*), the transmission was lower for *O. cornifrons* bees. Maccagnani et al. [41] found the European orchard bee, *O. cornuta*, carried up to 1000 times more *B. subtilis* than the honey bee and delivered a much higher dose to the flowers. We would expect a similar pattern (higher levels of transmission) with *O. cornifrons* nests. This transmission pattern could likely be due to the weak *O. cornifrons* nests at the time of this experiment. Weak nests of bees can influence the transmission rate of biological-control based products compared to strong nests where the number of bees is usually higher. However, it needs to be studied in future work.

Incubated plates from the controls showed there was no prior contamination of the crabapple trees used for the secondary transmission studies. Secondary transmission by *O. cornifrons* from flowers previously inoculated with *B. subtilis* to new unexposed flowers was successful (Table 1). The 24-h growth of *B. subtilis* colonies on these new flowers is demonstrated by the 6.7 to 61-fold increase in CFUs from that found on the primary transmission trees (Table 1). Successful secondary transmission and subsequent increases in *B. subtilis* colonies on flowers is very encouraging since it means the system is self-perpetuating. This attribute is likely to contribute to effective management of fire blight by timely controlling *E. amylovora* in commercial orchards. Refilling the nest dispensers with biological control product (*B. subtilis*, Serenade) on a daily basis may not be necessary and all pollinators visiting the inoculated flowers, not just *O. cornifrons*, should be able to move the *B. subtilis* colonies to new flowers as they open. Further work, however, is needed in managing strong nests of *O. cornifrons* in dispenser nests while they are deployed for vectoring. The dispenser and concept of using *O. cornifrons* as a vector works and hold promise for dispersing biocontrol agents for fire blight control. However, strong *O. cornifrons* nests would be needed to carry and deliver biological control products at high enough concentrations to give effective and optimal field control. Therefore, it is critically important to deploy a higher number of properly managed and healthy bees for vectoring biological control products. In addition, the foraging range of *O. cornifrons* is another important factor to consider while deploying them in commercial orchards, and the number of *O. cornifrons* nests per orchards should be adjusted based on the active foraging range of these bees and size of the orchards where they are to be deployed. The nest dispenser design could also be simplified and improved by eliminating the need for separate entrance and exit holes. Originally it was thought that *O. cornifrons* would not readily recognize and re-enter the dispenser nest through the transparent plastic grooves in the ramp, and require the opaque and black marked entrance tubes to enter the nest and would need to be redirected to separate exit holes/grooves in the plastic ramp that were much more easily re-filled with new control agent each day by pulling up the plastic flap over the grooves. Since we have shown the bees will readily use these exit holes also as for entrance, the design could be simplified by eliminating the entrance holes.

In recent past, fresh market demand for apples has led to widespread plantings of many new cultivars such as Gala, Fuji, and Honeycrisp and some traditional old cultivars (such as Jonathon and Rome), which are highly susceptible to fire blight disease [43]. These cultivars are highly dependent on bees and other insects for pollination during the bloom period. The Japanese orchard bees (*O. cornifrons*) and many other solitary bee species provide crucial ecosystem services in apple orchards with these and other commercial cultivars [44,45]. Therefore, deploying commercially available orchard bees such as *O. cornifrons* and *O. lignaria* for targeted delivery of biological control agents could be pivotal in the effective management of fire blight in apple and pear orchards and potentially other crops that are primarily dependent on biological control-based products for disease management.

## 4. Conclusions

This study demonstrates the feasibility of a newly developed nest dispenser for *O. cornifrons* bees for targeted delivery of biological control-based products for fire blight management. This study also demonstrates that *O. cornifrons* bees are effective in vectoring and delivering the biological control product (*Bacillus subtilis*, Serenade^®^) to apple blossoms, and are also capable of secondary inoculation of this biological control product to newly opened flowers. Future studies should develop this concept further and determine if other biological control agents (possibly more effective products) can also be vectored by *O. cornifrons* and other species of orchard bees. Such information would be useful in effective and timely management of fire blight in commercial organic apple orchards.

## Figures and Tables

**Table 1:** Primary and secondary transmission of *B. subtilis* by the Japanese orchard bee, *O. cornifrons*.

Transmission	Mean number of Colony-Forming Units (SE)
Primary Transmission *Trial 1*	6818 (4113)
Primary Transmission *Trail 2*	27,813 (11,428)
Secondary Transmission *Trial 1*	415,511 (218,531)
Secondary Transmission *Trial 2*	185,156 (129,613)

## Additional Information

As per the requirement of the funding, a project report (non-refereed publication) entitled “Development of the mason bee, *Osmia cornifrons*, as a targeted delivery system for biological control agents in the management of fire blight” was submitted to the State Horticultural Association of Pennsylvania (SHAP) by the PIs (D.B and H.N.), and it is made available to public and fruit growers via different internet resources and the Pennsylvania Fruit News prior to the refereed journal publication.
